# Clinical Effectiveness and Cost-Effectiveness of HIV Pre-Exposure Prophylaxis in Men Who Have Sex with Men: Risk Calculators for Real-World Decision-Making

**DOI:** 10.1371/journal.pone.0108742

**Published:** 2014-10-06

**Authors:** Anders Chen, David W. Dowdy

**Affiliations:** 1 Department of Internal Medicine, Johns Hopkins University School of Medicine, Baltimore, Maryland, United States of America; 2 Department of Epidemiology, Johns Hopkins Bloomberg School of Public Health, Baltimore, Maryland, United States of America; Centers for Disease Control and Prevention, United States of America

## Abstract

**Background:**

Oral pre-exposure prophylaxis (PrEP) can be clinically effective and cost-effective for HIV prevention in high-risk men who have sex with men (MSM). However, individual patients have different risk profiles, real-world populations vary, and no practical tools exist to guide clinical decisions or public health strategies. We introduce a practical model of HIV acquisition, including both a personalized risk calculator for clinical management and a cost-effectiveness calculator for population-level decisions.

**Methods:**

We developed a decision-analytic model of PrEP for MSM. The primary clinical effectiveness and cost-effectiveness outcomes were the number needed to treat (NNT) to prevent one HIV infection, and the cost per quality-adjusted life-year (QALY) gained. We characterized patients according to risk factors including PrEP adherence, condom use, sexual frequency, background HIV prevalence and antiretroviral therapy use.

**Results:**

With standard PrEP adherence and national epidemiologic parameters, the estimated NNT was 64 (95% uncertainty range: 26, 176) at a cost of $160,000 (cost saving, $740,000) per QALY – comparable to other published models. With high (35%) HIV prevalence, the NNT was 35 (21, 57), and cost per QALY was $27,000 (cost saving, $160,000), and with high PrEP adherence, the NNT was 30 (14, 69), and cost per QALY was $3,000 (cost saving, $200,000). In contrast, for monogamous, serodiscordant relationships with partner antiretroviral therapy use, the NNT was 90 (39, 157) and cost per QALY was $280,000 ($14,000, $670,000).

**Conclusions:**

PrEP results vary widely across individuals and populations. Risk calculators may aid in patient education, clinical decision-making, and cost-effectiveness evaluation.

## Introduction

In the United States, existing approaches to prevention have failed to control the HIV epidemic among men who have sex with men (MSM). Recent CDC data show that MSM accounted for 51% of prevalent cases of HIV and 61% of incident cases, despite representing only 2% of the overall population [1]. In 2010, results from the iPrEx clinical trial demonstrated the efficacy of once-daily emtricitabine-tenofovir disoproxil fumarate for oral pre-exposure prophylaxis (PrEP) to prevent HIV acquisition in MSM, with a relative risk reduction of 44% [2]. In 2012 emtricitabine-tenofovir disoproxil fumarate received FDA approval for PrEP, and in 2014, the CDC published clinical practice guidelines for PrEP use [3]. Multiple cost-effectiveness analyses have also been performed, and PrEP appears cost-effective on a population level at standard U.S. willingness-to-pay thresholds for those at high-risk for HIV infection [4]–[8]. However, PrEP uptake in clinical practice appears to be low to date [9], and questions have arisen around how to identify patients who should receive PrEP therapy [10]. Indeed, the clinical effectiveness and cost-effectiveness of PrEP are likely to vary highly across individuals and populations with different risk profiles. For example, some individuals on PrEP may engage in behavioral disinhibition (be willing to adopt riskier sexual behavior as a result taking PrEP) [11]–[13], and adherence to a daily preventive regimen may vary widely [2], [14], leading to different levels of clinical effectiveness. Similarly, PrEP cost-effectiveness will differ between geographic regions depending on baseline HIV prevalence and community antiretroviral therapy use.

Existing “global policy” models fail to capture and apply this heterogeneity to real-world decisions. Yet this information is available – clinicians often gain insight regarding patients' behaviors, and public health officials often have access to local epidemiologic data. A personalized risk calculator could improve individual estimates of PrEP clinical effectiveness and be useful for patient education, by demonstrating the positive or negative effects of certain behaviors [15], [16]. Likewise, a population-level risk calculator could improve estimates of PrEP cost-effectiveness and identify settings in which to focus deployment. Thus, we sought to create a model of HIV acquisition that could incorporate heterogeneity introduced from user-input parameters via online risk calculators, thus providing clinicians with estimates of the risk of HIV acquisition and clinical benefit of oral HIV pre-exposure prophylaxis for individual patients, and public health decision-makers with estimates of PrEP cost-effectiveness if deployed in their local populations.

## Methods

### Model Structure

We sought to create a model with a straightforward framework that would be easily understood and interpreted when used either as a personalized risk calculator in a patient-provider encounter or for rapid decision-making by policymakers without modeling expertise. We therefore constructed a decision analysis model that incorporates those parameters most likely to vary across patients and be estimable by providers or policymakers, including both non-modifiable factors (e.g. background HIV prevalence) and modifiable factors (e.g. PrEP adherence). Our primary outcomes were the number needed to treat (NNT) to prevent one HIV acquisition on the individual level and the cost per quality-adjusted life-year (QALY) gained on the population level. Whereas most global policy models have adopted a time horizon of 5–20 years of continuous PrEP use, clinical encounters and policy decisions in this high-risk population generally involve treatment decisions on a much shorter time frame. Thus, we modeled a 1-year duration of PrEP intervention costs and effectiveness to better reflect real-world patient-provider treatment plans. Similarly, we ignored secondary transmission (we included new HIV cases, but not future HIV cases attributable to the new cases), which is less relevant to clinical decisions for an individual patient. To assess the effects of these decisions, we compared our model against other more complex models that incorporated such effects. Our model can be downloaded from the risk calculator website (see below).

### HIV Acquisition

**Figure 1:**
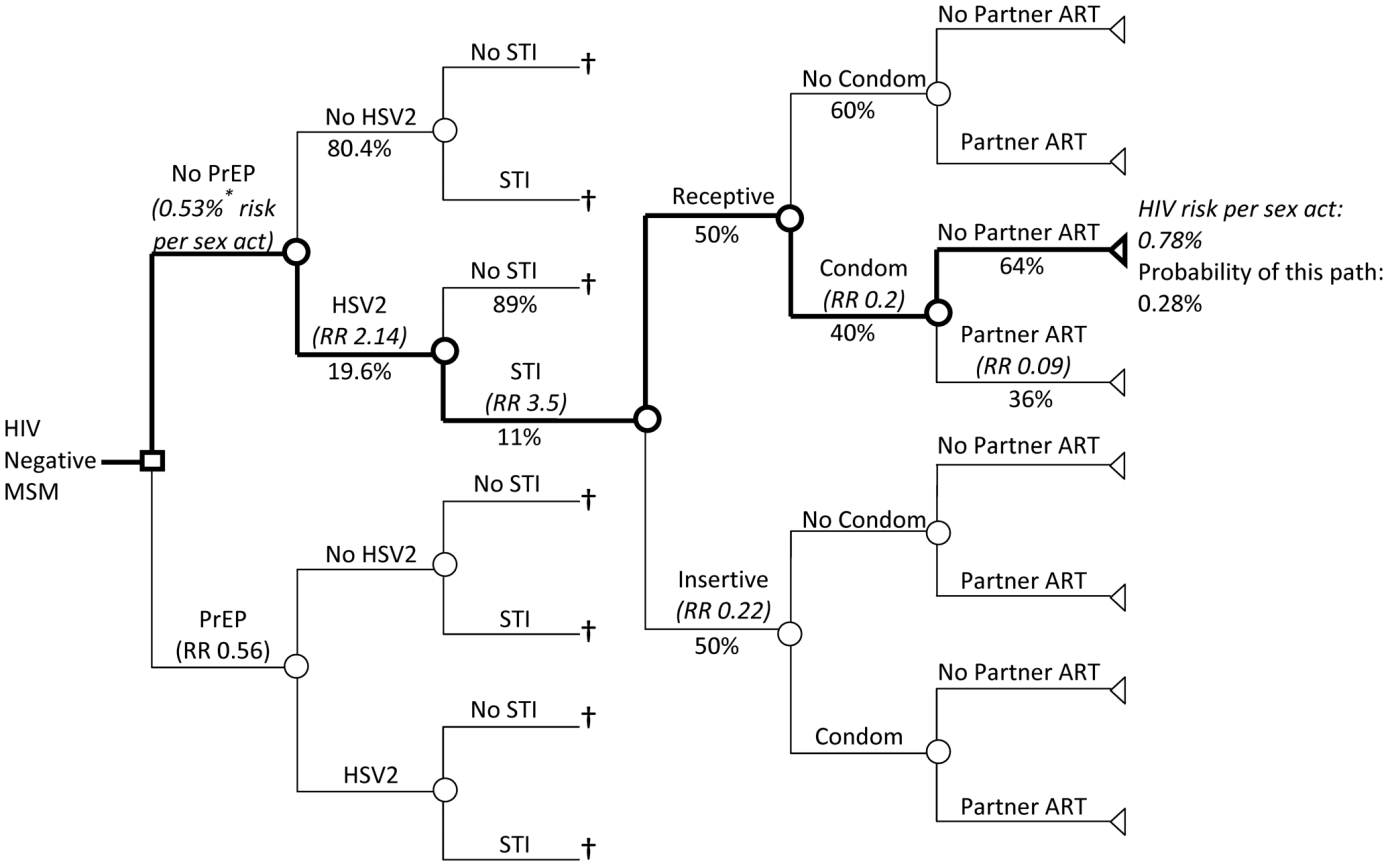
Decision tree comparing PrEP to No PrEP. Example path (bold) shows relative risk (RR) and probability for each node: for a sex act with no PrEP, where the partner is HSV2 positive and has an untreated STI, where the patient engages in receptive sex, uses a condom and the partner is not taking antiretroviral therapy (ART), the risk of HIV acquisition is 0.78% and the probability of this path is 0.28%. Overall risk per sex act is calculated using the cumulative risk and probability of all paths. Overall risk per month is calculated using estimates of sex acts per month and HIV prevalence (Table 1). Risk per year is calculated assuming exponential decay. ^*^ Literature estimate of 0.82% risk of HIV acquisition per receptive, unprotected anal sex act with HIV positive partner, without ART or PrEP (15). Stratifying by HSV2 and STI leads to 0.53% baseline risk. †For simplicity, branches 4, 5 and 6 and are only shown for one path. Abbreviations: HSV2: Herpes simplex virus-2; RR: relative risk; ART: antiretroviral therapy, STI: sexually transmitted infection.

The decision tree demonstrating the model structure is shown in **Figure 1**. Key model parameters are listed in **Table 1** [2], [5], [17]–[39]. We modeled HIV acquisition as an independent risk per unprotected receptive anal intercourse act with an HIV positive partner, as in prior studies [17], [40]. To this baseline acquisition risk we applied literature estimates of the relative risk and prevalence of modifying variables, including: insertive rather than receptive intercourse; antiretroviral therapy (ART) use in the infected partner; presence of herpes simplex virus-2 (HSV2), syphilis, gonorrhea, and chlamydia; condom use and PrEP use. Next we used estimates of HIV prevalence in the patients' community to calculate a per-act risk of HIV acquisition (i.e., regardless of a specific partner's HIV status). In this modeling framework, we made the simplifying assumption that all sex acts present an independent risk of HIV acquisition; we did not, for example, additionally model the number of partnerships or temporal heterogeneity in sexual behavior. This simplifying assumption allowed us to incorporate estimates of the monthly frequency of sexual activity [29] to estimate an individual patient's cumulative probability of HIV acquisition per month and per year. For the base-case scenario, epidemiologic parameters reflect generic US-wide estimates (e.g., from the Centers for Disease Control and Prevention, CDC); the corresponding risk calculators enable users to input model parameters most reflective of their specific circumstances.

**Table 1:** Parameter values for cost-effectiveness analysis.

Parameter	Value	Sensitivity Range	Reference
Risk of HIV acquisition			
Probability of HIV acquisition per sex act with HIV + partner^a^	0.0082	0.004–0.14	17, 40
Insertive anal sex act with HIV + person (relative risk)	0.22	0.1–0.3	17–19
HSV2 seropositive (relative risk)	2.14	1.5–3	20
HSV2 seropositive (prevalence)	0.196	0.05–0.4	21
ART (relative risk)	0.09	0.05–0.2	22–24
ART (prevalence)	0.36	0.2–0.6	25
PrEP (relative risk)	0.56	0.37–0.85	2
Condom use (relative risk)	0.2	0.1–0.3	26
Condom use (prevalence)	0.4	0.2–0.6	5
Untreated GC/CT/Syphilis (relative risk)	3.5	2–5	27
Untreated GC/CT/Syphilis (prevalence)	0.11	0.05–0.4	28
Average number of sex acts per month	7.06	5–10	29
HIV prevalence, MSM age 13–64	0.19	0.05–0.4	30
Costs, 2012 US$			
Annual cost of PrEP^b^	10,331	4,772–15,000	5
Lifetime cost per HIV patient, discounted^c^	305,521	150,000–500,000	5, 31–32
Average cost per case of STI treated (men)^d^	197	99–295	28, 33–34
Average cost per STI test	58	27–80	5
QALYs			
QALY gained per case of HIV averted, discounted^e^	2.24	1.07–3.2	5, 33
QALY lost per additional STI^f^	0.02	0.01–0.03	31, 34–37

a Risk per unprotected receptive anal sex act, with no ART use by the infected partner, and no PrEP use.

b Drug costs: $9,312; physician visits: $300; renal function tests: $13; HIV tests: $23.

c Annual cost of HIV care: $24,563; life expectancy 35 years.

d Cost per case of GC/CT/syphilis treated: $79/30/709; relative proportion of GC/CT/syphilis: 0.453/0.353/0.194.

e Disability weights for asymptomatic HIV/symptomatic HIV/AIDS: 0.94/0.82/0.7; years lived per stage of HIV infection: asymptomatic: 7, symptomatic: 21, AIDS: 7.

f Disability weight for symptomatic GC or CT/GC or CT epididymitis: 0.933/0.833; prevalence of symptomatic GC/CT: 0.31/0.28; prevalence of GC/CT epididymitis: 0.0069/0.0093; disability weights for primary/secondary/tertiary syphilis: 0.985/0.952/0.717; prevalence of primary or secondary syphilis/tertiary syphilis: 0.61/0.009; years disability with tertiary syphilis: 5.

Abbreviations: GC: Gonorrhea; CT: chlamydia trachomatis; QALY: quality-adjusted life-year.

### PrEP and other Modifying Variables

We compared a strategy of PrEP, following CDC guidelines [3], against no PrEP. PrEP includes documenting negative HIV status and adequate renal function prior to initiation, quarterly clinic visits and HIV testing, bi-annual screening for sexually transmitted infections (STI) and bi-annual renal function testing. PrEP efficacy is highly dependent on adherence; thus we modeled PrEP at differing levels of adherence as per iPrEx subgroup analyses [2]. We compared PrEP to no PrEP in a number of specific scenarios:

1. Base-case scenario (general MSM population): 44% PrEP efficacy, 19% background HIV prevalence, 40% condom use, no behavioral disinhibition.
2. Behavioral disinhibition (hypothetical scenario where PrEP use leads to riskier sexual behavior): 15% decrease in condom use, 15% increase in sexual encounters, and resulting 15% increase in STI prevalence among those taking PrEP.
3. High-adherence: 92% PrEP efficacy, reflective of iPrEx participants with detectable serum emtricitabine-tenofovir disoproxil fumarate drug levels [2].
4. High-risk: 35% background HIV prevalence.
5. High-risk and high-adherence: 35% background HIV prevalence and 92% PrEP efficacy.
6. Monogamous, serodiscordant relationship with partner ART use: 100% background HIV prevalence, 100% prevalence of partner ART use.
7. High condom use: 100% background condom use.

While we chose these scenarios for illustrative purposes, the corresponding risk calculators enable users not only to replicate these results, but also to explore a wide range of potential patient behaviors (clinical effectiveness) or population characteristics (cost-effectiveness).

### Economic Analysis

We used literature estimates to calculate costs and quality-adjusted life-years associated with each PrEP scenario vs. no PrEP, taking a societal perspective and lifetime analytic horizon. We estimated the cost of PrEP based on CDC recommendations for care (including drug costs, additional physician visits and laboratory testing beyond that which is recommended for persons not taking PrEP) and the existing literature [5]. We estimated the lifetime cost per additional case of HIV based on literature estimates of the annual cost of HIV care [31]. We also calculated the cost for the testing [5] and treatment [33] of additional sexually transmitted infections (gonorrhea, chlamydia and syphilis) for persons who engage in riskier sexual behavior (e.g. reduced condom use, more frequent sexual encounters) while on PrEP. For all costs, we adjusted to present value in 2012 U.S. dollars using the Medical Care component of the consumer price index [41], and applied a 3% discount rate for costs occurring beyond one year in the future [42]. We estimated QALYs associated with PrEP use based on literature estimates of life expectancy after HIV diagnosis [5], stage of infection [5], and disability weight associated with each stage [35]. We also calculated QALYs lost per additional case of STI in scenarios with behavioral disinhibition, using the disability weight [36] and the relative prevalence [37]–[39] of symptomatic urethral and epididymal gonorrhea/chlamydia; the disability weight [36] and the relative prevalence [33] of primary, secondary and tertiary syphilis; and the overall prevalence of gonorrhea, chlamydia and syphilis [28], [34]. We discounted QALYs for HIV and tertiary syphilis. We did not account for the possible reduction in quality of life stemming from emtricitabine- tenofovir disoproxil fumarate toxicity, or the development of drug resistant HIV, given the lack of evidence of either event in the iPrEx clinical trial.

### Personalized Risk Calculator

We created two online, interactive risk calculators coupled to our model of HIV acquisition, which can be accessed at the following websites:

Individual risk calculator: https://ictrweb.johnshopkins.edu/ictr/utility/prep.cfm

Population cost-effectiveness calculator: https://ictrweb.johnshopkins.edu/ictr/utility/prep2.cfm

The personal risk calculator is designed to allow providers to input specific values for certain parameters likely to vary by patient: (1) frequency of condom use, (2) frequency of insertive vs. receptive anal intercourse, (3) number of sexual encounters per month, and (4) current involvement in a monogamous serodiscordant relationship (with or without partner ART use). For patients not in monogamous serodiscordant relationships, the calculator also incorporates (5) the HIV prevalence in the patient's community, providing CDC data for HIV prevalence among MSM stratified by geography, race and age, and allowing for modification by the patient/provider in order to best estimate the patient's sexual network. Given data for these five model parameters, the risk calculator displays point estimates for the annual risk of HIV acquisition for 5 scenarios: (1) No PrEP, (2) PrEP at expected adherence (44% efficacy, as seen in the overall iPrEx study results), (3) PrEP with behavioral disinhibition (15% decrease in condom use, 15% increase in sexual frequency and 15% increase in STI prevalence), (4) PrEP with high adherence (92% efficacy), and (5) PrEP with high adherence (92% efficacy) plus 100% condom use.

The cost-effectiveness calculator allows users to input population-specific values for certain parameters: (1) HIV prevalence, (2) prevalence of antiretroviral therapy use, and (3) prevalence of condom use. It also allows for the evaluation of monogamous serodiscordant relationships, a distinct and important group of potential users of PrEP. The risk calculator displays point estimates for the cost per QALY gained with PrEP at expected adherence (44% efficacy), and PrEP at high adherence (92% efficacy), with the latter not being an alternative intervention per se, but rather a demonstration of how effective PrEP could be if adherence is high.

### Sensitivity and Uncertainty Analysis

We used the base-case model as a foundation for exploration of model variability and uncertainty in the results. For all model parameters, we performed both one-way sensitivity analysis and probabilistic uncertainty analysis, using point estimates and high/low estimates from the literature (**Table 1**). In the uncertainty analysis, we used Latin Hypercube Sampling to select values from beta distributions with shape parameter (alpha)  = 4 and minimum/maximum value as given in **Table 1**. We then calculated 95% uncertainty ranges as the boundaries of the 2.5^th^ and 97.5^th^ percentile from 10,000 simulations. We performed a three-way sensitivity analysis on three key model parameters (selected a priori as having particular influence on results): HIV prevalence, behavioral disinhibition, and PrEP adherence/efficacy. We describe the number needed to treat and cost-effectiveness at each point in a reasonable range of these three parameters.

### Ethics Statement

This study did not involve human or animal subjects and was not subject to the Internal Review Board/Ethics Committee.

## Results

### Individual-Level Benefit of PrEP

**Table 2** describes the projected impact of PrEP in a series of individual cases, as calculated by the personalized risk calculator. In the base-case scenario (patient fitting generic population profiles for MSM in the US), the NNT to prevent one HIV infection was 64 (95% uncertainty range: 26 to 176). In Scenario 2, a patient engaging in behavioral disinhibition, PrEP efficacy decreased from 44% to 28%, thus increasing the NNT to 97 (95% UR: 46 to 222). In Scenario 3 (patient with high adherence but population-average baseline risk and sexual behavior), PrEP efficacy increased from 44% to 92% (per iPrEx study results), and the NNT dropped to 30 (95%: UR 14 to 69). In Scenario 4, a higher-risk setting with 35% baseline HIV prevalence, the relative risk reduction did not change, but the absolute risk reduction increased, leading to a lower NNT of 35 (95% UR: 21 to 57). In Scenario 5 (high baseline HIV prevalence and high adherence), both factors increased the absolute risk reduction and lowered the NNT to 17 (95% UR: 10 to 27). In contrast, with Scenario 6 (monogamous serodiscordant relationship with partner ART use), the lower baseline risk led to a lower absolute risk reduction and a higher NNT of 90 (95% UR: 39 to 157). In Scenario 7, the lowest-risk scenario (100% condom use, not in a serodiscordant relationship), the NNT was much higher (212, 95% UR: 88 to 523).

**Table 2:** PrEP clinical effectiveness and cost-effectiveness in selected scenarios.

Scenario	Annual probability of HIV acquisition (95% UR)	PrEP relative risk reduction^a^	Number needed to treat^b^ (95% UR)	Cost per QALY gained, thousands of 2012 US$ (95% UR)
Base-case				
No PrEP	0.036 (0.015–0.087)			
PrEP	0.020 (0.008–0.054)	0.44	64 (26–176)	160 (CS-740)
PrEP, behavioral disinhibition^c^	0.026 (0.011–0.069)	0.28	97 (46–222)	320 (45-1,000)
PrEP, high adherence	0.003 (0.013–0.072)	0.92	30 (14–69)	3 (CS-200)
High-risk (35% HIV prevalence)				
No PrEP	0.065 (0.038–0.12)			
PrEP	0.037 (0.021–0.076)	0.44	35 (21–57)	27 (CS-160)
PrEP, high adherence	0.005 (0.003–0.10)	0.92	17 (10–27)	CS (CS-10)
Monogamous serodiscordant^d^				
No PrEP	0.026 (0.014–0.064)			
PrEP	0.014 (0.008–0.039)	0.44	90 (39–157)	280 (14-670)
100% condom use				
No PrEP	0.010 (0.004–0.025)			
PrEP	0.006 (0.002–0.017)	0.44	212 (88–523)	840 (230–2,500)

For non base-case scenarios, 95% uncertainty range was determined by holding the relevant user-determined parameters (e.g. condom use, PrEP adherence) fixed and conducting probabalistic uncertainty analysis with all other parameters.

Abbreviations: UR: Uncertainty range; CS: Cost saving.

a See Table 1 for sensitivity range for PrEP relative risk reduction.

b Number needed to treat to prevent 1 HIV infection.

c 15% decrease in condom use, increase in STI prevalence and increase in sexual frequency.

d Monogamous serodiscordant relationship with the HIV positive partner taking antiretroviral therapy.

### Population-Level Cost-Effectiveness of PrEP

**Table 2** also provides estimates of cost-effectiveness at the population level, as calculated by the population cost-effectiveness calculator. If PrEP were provided to a population representative of all MSM in the US, the cost per quality-adjusted life-year (QALY) gained was $160,000 (95% UR: cost saving to $740,000). Behavioral disinhibition (scenario 2) made PrEP less cost-effective, at $320,000 per QALY gained (95% UR: $45,000 to $1,000,000), whereas high adherence improved its cost-effectiveness, to $3,000 per QALY gained (95% UR: cost saving to $200,000). In settings with high baseline HIV prevalence (e.g. 35% in Baltimore, Maryland; Scenario 4), cost-effectiveness also improved from the baseline to $27,000 (95% UR: cost saving to $160,000), and in populations with both high HIV prevalence and high adherence (Scenario 5), PrEP was cost saving (95% UR: cost saving to $10,000 per QALY gained). The cost per QALY gained was highest in scenarios 6 (monogamous serodiscordant relationships with partner ART use) and 7 (100% condom use), at $280,000 (95% UR: $14,000 to $670,000) and $840,000 (95% UR: $230,000 to $2,500,000) respectively.

### Sensitivity Analyses

**Figure 2:**
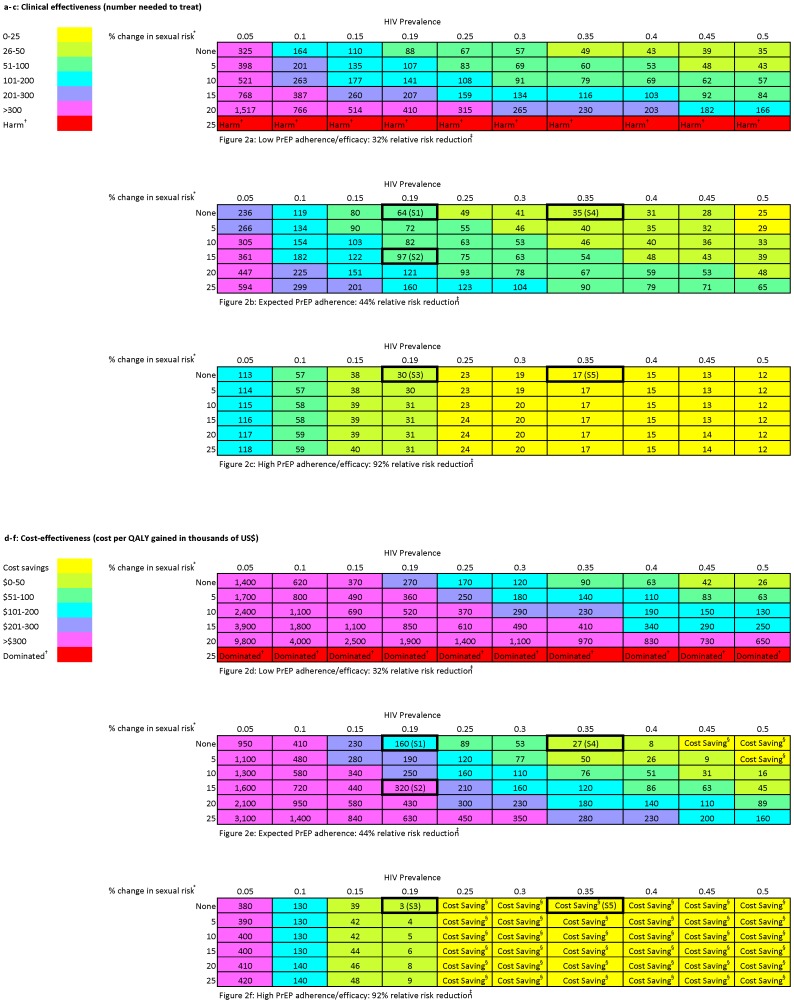
Clinical effectiveness and cost-effectiveness as a function of PrEP adherence, HIV prevalence and behavioral disinhibition. * Percent decrease in condom use, increase in STI prevalence and increase in sexual frequency. † Harm refers to clinical scenarios where the intervention leads to an increase in HIV acquisition. Dominated refers to cost-effectiveness scenarios with higher cost and worse outcomes. ‡ Low, expected and high adherence/efficacy refers to the relative risk reduction seen in 3 iPrEx subgroups: <50% reported pill use (low), the overall study (expected), and the subgroup with detectable serum drug levels (high) (2). § Cost Saving refers to scenarios with lower cost and better outcomes. S1–S5 denote Scenarios 1–5 from the text.

To complement the risk calculators, we performed a three-way sensitivity analysis to provide a more comprehensive visual description of the clinical effectiveness (**Figure 2a–c**) and cost-effectiveness (**Figure 2d–f**) of PrEP across a range of values for 3 key parameters: background HIV prevalence (5% increments), behavioral disinhibition (5% increments) and PrEP adherence (3 discrete levels, per iPrEx data). Scenarios 1–5 above are marked S1–S5 to demonstrate where they fit within this range. For any patient scenario with low PrEP adherence (**Figure 2a**), the NNT exceeded 50 unless the HIV prevalence was high (at least 35%) and behavioral disinhibition was low (less than 10% change in sexual risk). At low adherence and high behavioral disinhibition, PrEP was harmful, leading to an increased risk of HIV acquisition. For clinical scenarios with expected levels of PrEP adherence (**Figure 2b**), the NNT exceeded 50 for HIV prevalence levels below 25%. If patients took PrEP with high adherence (**Figure 2c**), the NNT was less than 50 in any setting where HIV prevalence was 15% or higher. With high adherence, the effect of behavioral disinhibition was greatly attenuated: at the national average of 19% HIV prevalence, the difference in the NNT between zero and 25% behavioral disinhibition was 1, compared to a difference of 96 with expected adherence and 322 with low adherence.

In populations where PrEP adherence was low (**Figure 2d**), the cost per QALY gained exceeded $100,000 for all scenarios except those with high HIV prevalence (at least 35%) and low behavioral disinhibition (less than 10% change in sexual risk). At low adherence and high behavioral disinhibition, no PrEP was less costly and more effective than (i.e., dominated) PrEP. At expected levels of PrEP adherence (**Figure 2e**), the cost per QALY gained exceeded $100,000 for HIV prevalence levels below 25%. At expected adherence, cost-effectiveness was highly dependent on the degree of behavioral disinhibition. For example, at 45% HIV prevalence, PrEP ranged from cost saving (no behavioral disinhibition) to $200,000 per QALY gained (25% increase in risky sexual behavior). For populations with high adherence (**Figure 2f**), PrEP was cost saving at HIV prevalence above 21%. As with clinical effectiveness, behavioral disinhibition had little impact on cost-effectiveness when PrEP was taken at high adherence.

**Figure 3:**
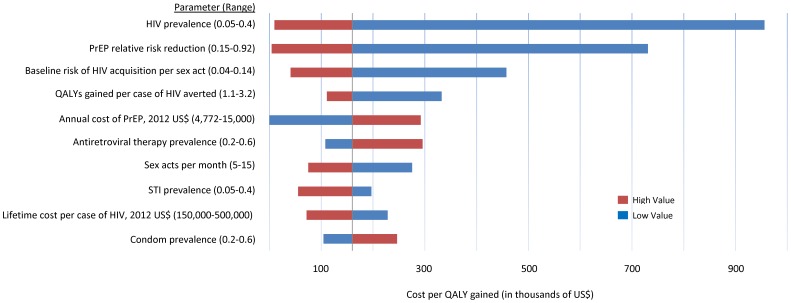
One-way sensitivity analysis of PrEP cost-effectiveness (in thousands of US$). The black vertical line represents the base case-scenario relative to no PrEP ($160,000 per QALY gained). Blue bars represent the low value of the range, and red bars represent the high value of the range. Bars to the left of the base case scenario represent more favorable scenarios. Only parameters which affected the cost-effectiveness ratio by more than 50% in either direction are shown.

One-way sensitivity analyses of key parameters (for which variation changed cost-effectiveness estimates by +/−50%) are shown in **Figure 3**. In addition to HIV prevalence, PrEP efficacy and the individual components of behavioral disinhibition (prevalence of condom use, STI prevalence, and frequency of sexual activity), other parameters with the highest impact on cost-effectiveness were the baseline risk of HIV acquisition per sex act, QALYs gained per case of HIV averted, and PrEP annual cost. If the annual cost of PrEP was reduced by roughly 50% to below $4772, PrEP was cost saving in the base-case scenario.

## Discussion

This decision model suggests that the effectiveness and cost-effectiveness of oral PrEP is highly dependent on condom use, HIV prevalence, PrEP adherence, and the degree of behavioral disinhibition – factors which vary widely between patients and populations. For example, PrEP is cost-saving in individuals with high-risk and high adherence, yet unlikely to have an important effect for patients who are already using condoms 100% of the time (NNT  = 212, $840,000 per QALY gained). Physicians must take these patient-level factors into account when deciding whether to prescribe PrEP, and public health practitioners must take these population-level factors into account when deciding where to focus public health resources. Existing models cannot inform these types of decisions. Our model and interactive risk calculators may help close this knowledge gap.

Our model uses a simpler structure than others in the literature, both to facilitate translation to a risk calculator and to represent real-world decisions (e.g. the shorter duration of PrEP use). To test our model performance, we used baseline parameters from the literature and compared our model results to those from a widely-cited cost-effectiveness analysis by Juusola and colleagues [5]. We report a base-case cost per QALY gained of $160,000, compared to their estimate of $172,000. In our high-risk subgroup, we report a cost per QALY gained of $27,000, similar to their corresponding high-risk estimate of $40,000. Our model estimates of HIV acquisition also correspond well with existing estimates from the primary literature. Our base-case estimate of the annual probability of HIV acquisition without PrEP is 3.6%, and with PrEP is 2% – nearly identical to results from the iPrEx clinical trial at 52 weeks [2]. We note that the uncertainty ranges in our analysis are larger than those in prior analyses, reflecting the fact that uncertainty is greater at an individual level than at a population-average level.

This model offers, for the first time, personalized estimates of PrEP clinical effectiveness. We also include the effect of STI prevalence and behavioral disinhibition on PrEP – concerns that often play a role in patient-provider interactions and which have been underemphasized in policy-level models of PrEP. By varying parameters under the patient's control, providers can use the personal risk calculator for patient education by demonstrating the effects of behavior modification on the risk of HIV acquisition. Proactive reinforcement of PrEP adherence, condom use and other non-pharmacologic HIV prevention strategies will be critical in maximizing PrEP effectiveness; our calculator can serve as an important tool in this process.

This model also offers a customizable cost-effectiveness calculator to aid in public health and population-level decisions. HIV prevalence and antiretroviral therapy use vary widely across geographical regions in the United States, and this tool will allow local-level evaluation of PrEP strategies. This analysis also offers quantitative evaluation of monogamous serodiscordant relationships, which is of special interest given the relative ease of reaching and treating this population. For those whose partners are on antiretroviral therapy, our results demonstrate that the cost-effectiveness of additional PrEP use is low, and that resources may be better used to target other, higher risk patients. It should be noted that not all stated monogamous, serodiscordant relationships are truly monogamous, and our analysis does not account for such discrepancies.

As with any modeling study, our analysis has limitations. Our model has not yet been prospectively validated with cohort data. We believe this is a critical next step, and present our model and risk calculator to make available for other teams to potentially evaluate in a broad array of populations, with the belief that this can be a faster path to wide validation than only presenting summary data. While awaiting such validation, we believe that making the risk calculator available to clinicians is appropriate, given there is no other existing tool, and given the model's strong performance compared to actual results seen in the iPrEx cohort (from which only one of our model parameters was derived). The model framework also makes simplifying assumptions regarding HIV partner networks and does not account for factors such as assortative mixing. The risk calculators also cannot capture a complete picture of individual risk – only certain parameters can be entered by users, and even these entries (e.g. self-reported frequency of condom use) will not perfectly estimate the risk involved. However, our model performs well compared to clinical data and existing models, and the risk calculators offer a balance between an acceptable level of complexity for the user interface and a powerful predictive tool to aid in patient education and decision-making. As with any risk calculator, it is only a tool, and can only provide a starting point for providers and patients to engage in discussion and shared decision-making. Risk may also change over time as patient behaviors change, and frequent provider contact, as recommended by CDC guidelines, will be needed to ensure an accurate clinical picture. As with any cost-effectiveness analysis in the US, our study may have limited impact in regards to coverage decisions and national bodies such as Medicare and the United States Preventative Services Task Force. However, our intent is not to influence such decisions, but rather to aid local and state health departments in deciding whether to support, implement and fund PrEP initiatives. Finally, our model is not representative of MSM in low-income countries (where our unit cost estimates will not be accurate), or of other risk groups including heterosexual men, women or injection drug users.

In summary, the risk of HIV infection and the degree of PrEP effectiveness vary widely across patients and populations, and existing studies do not provide adequate aid in incorporating this variation into real-world decision-making. We present a novel model of HIV acquisition linked to interactive, online risk calculators that provide customized estimates of PrEP clinical effectiveness and cost-effectiveness. Our findings extend the existing literature base that suggests PrEP can be cost-effective in high-risk MSM or when adherence is high, demonstrating quantitatively the patients and populations for whom clinical effectiveness and cost-effectiveness are likely to be maximized. Although national-level policies are a necessary first step, PrEP programs are likely to be implemented at a local level, and ultimately, PrEP can only be used to prevent HIV on a patient-by-patient basis. Our model can serve as an important tool in these processes, enabling public health practitioners to identify high-risk populations, and enabling clinicians to educate patients, encouraging them to adopt a comprehensive set of behaviors – including PrEP – that will minimize their risk of acquiring HIV and thereby optimize their health and well-being.
